# Consensus recommendations for the assessment and treatment of perinatal obsessive–compulsive disorder (OCD): A Delphi study

**DOI:** 10.1007/s00737-023-01315-2

**Published:** 2023-05-03

**Authors:** Melissa Mulcahy, Christian Long, Teagan Morrow, Megan Galbally, Clare Rees, Rebecca Anderson

**Affiliations:** 1 Discipline of Psychology, School of Population Health, enAble Institute, Faculty of Health Sciences, Curtin University, Perth, WA Australia; 2 College of Science, Health, Engineering and Education, Murdoch University, King Edward Memorial Hospital for Women, WA Perth, Australia

**Keywords:** Perinatal mental health, Postpartum, Obsessive–compulsive disorder, Psychiatric assessment and treatment, Best practice, Clinical standards and guidelines

## Abstract

The perinatal period is one of increased vulnerability to parents experiencing the onset of, or an increase of existing, obsessive–compulsive disorder (OCD) symptoms. Existing OCD and perinatal mental health best practice guidelines do not detail specific considerations relevant to OCD in the perinatal period (‘Perinatal OCD’). Perinatal OCD risks being undiagnosed or misdiagnosed, and subsequently untreated or mistreated, with potential negative impacts for individuals and families experiencing this problem, highlighting the importance of specific guidance. This study employed a modified Delphi survey methodology to establish recommended best practice for the assessment and treatment of perinatal OCD. A literature review identified 103 initial best practice recommendations, and participants suggested 18 further recommendations. These recommendations were rated for importance over three survey rounds by two expert panels, comprising of 15 professionals with clinical or research expertise in perinatal OCD and 14 consumers with lived experience of perinatal OCD. One-hundred and two statements were endorsed for inclusion in the final set of recommendations for clinical best practice with perinatal OCD. These recommendations inform practice across eight themes; psychoeducation, screening, assessment, differential diagnosis, case care considerations, treatment, partners & families, and culture & diversity. This novel study is the first to collate and outline a set of clinical best practice recommendations, developed using the consensus perspectives of both individuals with lived experience and professionals with relevant expertise, for supporting individuals with perinatal OCD and their families. Differences between panel perspectives, and directions for future research are also discussed.

**Supplementary Information:**

The online version contains supplementary material available at 10.1007/s00737-023-01315-2.

## Introduction

The perinatal period has been identified as a period of increased vulnerability to the onset or exacerbation of obsessive–compulsive disorder (OCD), particularly among women (Russell et al. 2013). Perinatal obsessions and/or compulsions often reflect an anxious preoccupation with the safety or wellbeing of the foetus or infant, or with one’s responsibility to prevent harm as a primary caregiver (Abramowitz et al. 2003; McGuinness et al. 2011). For example, individuals with perinatal OCD (pnOCD) often experience intrusive and distressing thoughts (‘obsessions’) about the foetus/infant being harmed in some way, or about being unable to care appropriately for their baby (Fairbrother & Abramowitz 2016). Certain obsessive–compulsive symptoms (OCS), including harming and sexual obsessions appear to be more common when OCD occurs in the postpartum period, compared with OCD in pregnancy or other life periods (Starcevic et al. 2020). Other common symptoms of OCD in the general population, including washing/cleaning compulsions, appear to be less frequent in the postpartum. Studies indicate that between 2.2% and 16.9% of women experience OCD in the postpartum (Fairbrother et al. 2016, 2021a, b; Fawcett et al. 2019; Osnes et al. 2019; Russell et al. 2013), with the higher prevalence rates reported in more recent studies most likely being attributable to broadening of the criteria for OCD in the *Diagnostic and Statistical Manual of Mental Disorders: Fifth Edition* (American Psychiatric Association, 2013; Fairbrother and Collardeau, 2021).

The increased prevalence, distinctive symptom presentation, and potentially unique impacts associated with OCD (McGuinness et al. 2011; Russell et al. 2013) in the perinatal period indicates the need for specific guidelines to inform best-practice in working with new/expecting parents with OCD. Comprehensive clinical guidelines for the assessment, treatment and management of OCD have been published (American Psychiatric Association, 2007a,b; National Institute for Health and Care Excellence [NICE], 2005), but lack specific guidance for OCD in the pre- and postnatal periods (Fairbrother & Abramowitz 2016; McGuinness et al. 2011; Russell et al. 2013). The American Psychiatric Association (APA) guidelines on OCD references treatment considerations during pregnancy and breastfeeding but focuses on pharmacotherapy (e.g., medication risks and side effects) rather than psychological considerations (American Psychiatric Association, 2007a, b).

Several broader guidelines exist for psychological considerations and/or treatment during the perinatal period, such as those from beyondblue (2011), the Centre of Perinatal Excellence (Austin and Highet 2017), and the National Institute for Health and Clinical Excellence (National Institute for Health and Care Excellence 2014Such guidelines are valuable for a general understanding of mental health care in the perinatal period (Austin and Highet 2017; beyondblue 2011; National Institute for Health and Care Excellence 2014); however, guidance on OCD is typically subsumed within anxiety disorder recommendations. Although OCD has been classified as an anxiety disorder (Stein et al. 2010), there is growing recognition that OCD has distinctive features, requires unique diagnostic (American Psychiatric Association, 2013) and treatment perspectives (McKay et al. 2015; Stein et al. 2010), and thus warrants specific guidance. The lack of development of pnOCD-specific clinical guidelines reflects a dearth of empirical research on the treatment and management of the disorder. Nonetheless, a small number of studies have provided evidence supporting pharmacological (Misri et al. 2004; Misri & Milis 2004; Sharma 2018) and psychological treatment for pnOCD (Challacombe et al. 2017; Challacombe & Salkovskis 2011; Christian & Storch 2009; Gershkovich 2003; Misri et al. 2004; Puryear & Treece 2017).

The current study aims to address this gap in the clinical literature on perinatal OCD, by systematically developing clinical practice recommendations for assessing, managing, and treating pnOCD, and supporting individuals with this disorder and their families. We used a Delphi survey methodology to collate the views of professionals with clinical and/or research expertise in, as well as consumers with lived experience of, pnOCD. We intend that the recommendations resulting from this study may be used to inform best-practice clinical care for individuals with pnOCD, and to increase health practitioners’ understanding of this disorder and its treatment.

## Method

### Research design

The current study used a Delphi survey methodology to identify best practice recommendations for the assessment and treatment of individuals with pnOCD. The Delphi technique is widely used in health research to systematically ascertain a reliable consensus from a group of experts to inform service planning and practice (Jorm 2015). It is done via a series of survey rounds in which each member of the expert panel individually rates the extent to which they consider recommendation statements to be important. The current study utilised a modified Delphi methodology as described by Keeney et al. (2010) and comprised two panels; consumers as experts in their lived experience with pnOCD (i.e., the Consumer panel), and clinicians (i.e., psychologists, psychiatrists, researchers) specialising in pnOCD (i.e., the Professional panel). Ratings were made over three successive survey rounds, in accordance with endorsement criteria detailed in Table 1. During the first survey round, panellists were given the opportunity to suggest statements to be added or amended for the next round. In each subsequent round, participants were provided with a summary of how both panels responded in the previous round to each statement being re-rated, as well as how they, individually, responded to the statements.

**Table 1:** Statement endorsement criteria over 3 review rounds

Round	Endorsed	Retained for re-rating	Rejected
Round 1	≥ 80% by both panels	≥ 80% by one panel but < 80% by other panel, or 70–79.9% by both panels	< 70% by both panels
Round 2	≥ 80% by both panels	≥ 80% by one panel but < 80% by other panel, or 70–79.9% by both panels	< 70% by both panels
Round 3	≥ 80% by both panels	Not applicable	< 80% by either panel

Endorsement is based on the percentage of ‘essential’ or ‘important’ ratings.

### Participants

Recruitment of the Consumer panel occurred by contacting relevant consumer advocacy/support services in Australia, United Kingdom, and the United States of America. Consumer panellists (*n* = 18) were English-speaking females over 18 years of age, who reported living with, or having previously experienced, OCD during the perinatal period, diagnosed by a mental health professional. Consumers were deemed ineligible to participate in the study if, based on a pre-study measure of OCD severity, they reported ‘severe’ current symptoms on a measure of OCD severity (see ‘Measures’ section below). This was due to concerns their severity may impact insight and/or that reflecting on their care needs while experiencing severe symptoms could add to their distress. Potential participants for the Professionals panel (*n* = 20) were identified via relevant professional organisations (e.g., the International Marcé Society), perinatal mental health LISTSERVs, provider registers, authors of relevant pnOCD publications found via a literature review, and via the professional networks of the study researchers. Participants were invited to be a part of the Professionals panel if they had at least five years of relevant experience working with individuals with perinatal mental health disorders, including assessing and treating pnOCD, and/or had published peer-reviewed academic publications on pnOCD. A five-year minimum was set to ensure that professionals had adequate experience and expertise with pnOCD.

For homogeneous Delphi samples it is recommended that a minimum of 10–15 participants be used to ensure reliability of the survey results (Keeney et al. 2010). A total of 14 Consumer panellists and 15 Professional panellists completed the first survey round and, thus, were included in the final study sample. The retention of participants who responded to the first round ensured that the consensus reached in later rounds was not biased by participants dropping out of either panel (Keeney et al. 2010).

### Materials

*Consumer symptom severity screening.* The 10-item *Yale Brown Obsessive–Compulsive Scale – Self Report version* (*YBOCS-SR;* Fineberg 2001) was used to screen potential members of the Consumer panel for OCD symptom severity prior to the survey rounds. Previous studies have found the *YBOCS-SR* to have strong internal consistency, test–retest reliability, and construct validity (Storch et al. 2011). A total summed score of < 24, corresponding with ‘mild’ or ‘moderate’ symptoms, was the cut-off for inclusion in the study.

*Initial statement formation.* Prospective guideline statements were created via review of the existing literature. Specific search terms included: perinatal obsessive–compulsive disorder, postpartum obsessive–compulsive disorder, perinatal OCD, postpartum OCD, postnatal OCD, pregnancy OCD, maternal OCD, antenatal obsessive–compulsive disorder, postnatal obsessions, postpartum obsessions, OCD best practice, and perinatal best practice. Databases searched included PsycArticles (Ovid), PsychINFO (Ovid), Psychiatry online, Science Direct, PubMed, ProQuest, Taylor & Francis, Google Scholar, and Cochrane Library. Existing OCD (American Psychiatric Association, 2007a, b; National Institute for Health and Care Excellence, 2005) and perinatal mental health guidelines (Austin and Highet 2017; beyondblue 2011; National Institute for Health and Care Excellence 2014) were also reviewed. Additionally, panel members were invited to submit information they thought should be included in the guidelines via open text submissions on Qualtrics Survey Software.

The proposed statements and suggestions from panellists were reviewed by a research team for relevance, clarity, and repetition of content. The research team comprised one perinatal psychiatrist (M.G.) and two senior clinical psychologists with clinical and research expertise in pnOCD (R.A. & C.R.), a doctoral student (M.M.), and two postgraduate masters students (C.L. & T.M.) conducting research on pnOCD. A total of 103 initial statements were generated and organised by topic. To support the generation of evidence-based guidance, a plain language summary of the current research literature on clinical approaches to pnOCD was created and provided to participants. The clinical literature summary was also reviewed by the research team for content and clarity.

### Procedure

Ethics approval was obtained from the Curtin University Human Research Ethics Committee (No. HRE 2017-0087) before study commencement. The recommendation statements formed by the research team were presented to panellists for rating over three survey rounds. The statements, along with definitions of key terms and the clinical literature summary, were presented online via Qualtrics. During each round, panellists chronologically reviewed and rated each statement using a Likert scale; ratings of 1 (‘essential’) and 2 (‘important’) indicated endorsement of the item, and 3 (‘don’t know/depends’), 4 (‘unimportant’), and 5 (‘should not be included’) considered to be not endorsed. Panellists were instructed to respond based on any knowledge available to them, including clinical or lived-experience, research evidence, or any other experience related to perinatal OCD. They were also asked to consider the clinical literature summary when completing each round so that the recommendations would reflect evidence-based practice and practice-based evidence provided by experts (Jorm 2015).

In Round 1, panellists were able to write-open ended responses regarding any additional statement suggestions, or amendments to existing statements, they felt necessary. Any additional recommendation statements generated by participants were reviewed by the research team and included for rating by the panels in Round 2. Consensus criteria for whether each statement is considered endorsed, retained for re-rating, and not endorsed is available in Table 1. Those recommendations that were endorsed or rejected in previous rounds were not included in subsequent rounds. Participants were given two weeks to complete each round, with up to three reminder emails sent to participants yet to complete the round, and they were able to return to the survey form to complete the round at a later time if they were unable to do so in one sitting. Participants who completed at least 50% of Round 1 were invited to respond to Round 2 and 3. They were asked to consider the following information provided to them about the statements from the previous round retained for re-rating: how they previously responded to each item, and the level of endorsement of the item by each panel and overall. Statements considered ‘endorsed’ after the three rounds were included and those ‘not endorsed’ were excluded from the final recommendation set. This list of final recommendations grouped by topic area can be found in Table 2.

**Table 2:** List of endorsed statements for final recommendation, by topic

Topic	Statement
Psychoeducation	General information provided to expectant and new parents about perinatal mental health and wellbeing should include PnOCD.^a^ Psychoeducation should be provided to normalise the experience of intrusive thoughts in the general population Psychoeducation should be provided to normalise the increase or onset of intrusive thoughts in the perinatal period Psychoeducation should be provided to explain that intrusive thoughts may be more frequent during the perinatal period for a range of reasons (e.g., lack of sleep, role transitions, increased feelings of responsibility, stress) Psychoeducation should be provided to normalise that the perinatal period is one of significant life changes, that perinatal mental health concerns are common during this period, and that treatments are available.^a^ Psychoeducation should be provided explaining that in OCD intrusions are experienced as distressing as they are unwanted and inconsistent with the parent’s true values and desires Psychoeducation should be provided that normal, everyday intrusions can become OCD when parents worry about the intrusions representing their true values and desires, or that their intrusions might influence them to act against their values, or that their intrusions need to be responded to with behaviours aimed at restoring a sense of safety or certainty (e.g., cleaning, checking) Psychoeducation should be provided that there is no evidence that parents experiencing harm-related intrusions, no matter how horrific in content, will act on these thoughts Common themes of perinatal obsessions and/or intrusive thoughts, including fear of contamination, harm (occurring to the baby or others), violence, perfectionism, and uncertainty about something which the parent did or did not do should be discussed with the parent/s Common themes of perinatal compulsions, including checking behaviour, reassurance seeking, rituals, mental neutralising efforts (e.g., praying, distraction), and avoiding feared situations or activities should be discussed with the parent/s Psychoeducation about pnOCD should be provided by front line perinatal healthcare workers such as General Practitioners, Obstetricians, Midwives, and Child/Maternal Health Nurses Parents should be informed that there are effective treatment options available for pnOCD and, if left untreated, can become chronic and continue to impact on the parent’s relationship with the child and other family members When parents are informed about effective treatments for PnOCD, a description of both psychological and pharmacological treatment options should be provided With the individual’s permission, psychoeducation about pnOCD should be provided to significant others
Screening	People with a history of mood, anxiety or OCD symptoms should be routinely screened for PnOCD.^a^ Screening tools used for detection of perinatal mental health should include items specific to pnOCD If a parent does not consent to pnOCD assessment, the health professional should document this, and they should be offered the opportunity to request an assessment in the future should they change their mind.^a^ Health practitioners should be aware that many parents may decline an assessment or not be forthcoming about their PnOCD symptoms due the parents’ anxious concerns that disclosure will result in child safeguarding When parents are informed about effective treatments for PnOCD, a description of both psychological and pharmacological treatment options should be provided Health professionals must be able to arrange follow-up assessment and/or care if there are concerns for the safety of the parent, the fetus/infant, or other children in their care as a result of pnOCD, that is consistent with relevant legislation and practice standards
Assessment	At the outset of an assessment of pnOCD symptoms, the parent should be provided with an explanation of the assessment, explaining the routine nature of the assessment and the limits of confidentiality, and provide their informed consent.^a^ PnOCD assessment should be undertaken by trained mental health professionals with the relevant skills and knowledge to conduct the assessment, determine the level of required support, provide health promotion and refer to appropriate services if relevant Mental health professionals should utilise the assessment process to normalise the symptoms of pnOCD and provide psychoeducation around the OCD cycle and common features Mental health professionals should provide a list a common intrusions and compulsive behaviours in the perinatal period to help parents identify symptoms and prompt disclosure Mental health professionals should ask directly about the presence of any intrusive thoughts/obsessions and/or compulsions Mental health professionals should directly ask about, while also normalising, the presence of taboo intrusions such as thoughts that they might physically or sexually harm their child despite having no wish or intention to do this Mental health professionals should enquire about pnOCD related behaviours designed to reduce distress and restore a sense of safety such as excessive avoidance (e.g., avoiding breastfeeding the baby), reassurance seeking, mental compulsions (e.g., praying), and thought suppression/distraction Assessment of pnOCD symptoms should cover the pervasiveness, intensity, frequency, duration of, insight to and resistance of obsessions and compulsions, and the level of distress and impairment these cause Assessment should consider the course of any symptoms, specifically, whether any OCD symptoms or disorder existed prior to perinatal period and if they have changed since becoming pregnant/having the baby, and any changes in symptoms or severity Assessment should explore the subjective and objective impacts of pnOCD on caregiving behaviours, relationships with significant other/s, and on other activities of daily living Assessment of pnOCD should consider previous or concurrent/comorbid mental health diagnoses and treatment response Assessment should consider and document if there are concerns for the safety of the parent, the fetus/infant, or other children in their care as a result of pnOCD and arrange follow-up assessment and/or care that is consistent with relevant legislation and practice standards The parent should be allowed to decide who is present during the pnOCD assessment (alone, with significant others, with children etc.).^a^
Differential Diagnosis	Clinicians should determine whether any taboo thoughts present are experienced as ego-syntonic (consistent with the parent’s beliefs, desires and wishes) or ego-dystonic (inconsistent with the parent’s beliefs, desires and wishes and therefore experienced as senseless, unwanted and intrusive) Clinicians should determine whether parents are misinterpreting physiological signs of anxiety as evidence of sexual arousal or aggression Clinicians should determine whether parents are attempting to avoid or suppress intrusive thoughts (indicative of pnOCD) versus engaging in the thoughts with an increasing amount of detail (indicative of other mental health concerns) Clinicians should differentiate pnOCD fears that are bizarre and senseless (e.g., contaminating the baby by nappy changing), from depressive ruminations that are typically sad or pessimistic thoughts about themselves, the world and the future (e.g., “I’m an inadequate parent”) and from delusions, which represent fixed false beliefs which are accepted by the parent as being self-evidently true (e.g., “The FBI is coming to take my baby”) Clinicians should differentiate pnOCD behaviours that are marked by avoidance, thought suppression and reassurance seeking, from behaviours that indicate other mental health concerns (e.g., withdrawal/amotivation associated with depressed mood, lack of goal directed activity associated with schizophrenia) Clinicians should determine whether parents are avoiding triggers that increase intrusive thoughts (indicative of pnOCD) versus seeking out stimuli that would allow them to think more about these taboo thoughts (indicative of other mental health concerns) Clinicians should assess the impact of any psychosocial issues that may play a role in the onset and maintenance of pnOCD (e.g., easy vs. unsettled baby, family support, social support) Clinicians should assess the impact of any physical and psychological issues that may play a role in the onset and maintenance of the pnOCD (e.g., issues with breast feeding, pregnancy complications, difficulties with delivery, wanted/unwanted birth, pregnancy related anxieties, issues related to transition to parenthood, comorbidities), and the implications for treatment Clinicians should assess the impact of any pnOCD symptoms on activities of parenting (e.g., breast feeding, bathing child, engaging in play with child) Health professionals such as General Practitioners, Community Nurses, Midwives, Psychologists, and emergency department staff should have regular education and training in perinatal MH, that includes specific focus on pnOCD and its differentiation from other common perinatal MH conditions In cases of differential diagnosis uncertainty, another clinician with specific expertise in pnOCD assessment and management should be consulted Assessments and response to treatment should be documented by the pnOCD clinician and information shared, with the patient’s consent, with other health professionals providing ongoing care (e.g., General Practitioner, Psychologist or Psychiatrist, Midwives, Obstetrician, Nurses) Referral for child safeguarding should, where possible, only be made following a thorough differential diagnostic assessment by an experienced pnOCD clinician
Case Care Considerations	Organisations should have a clear policies and procedures regarding treatment, referrals, collaboration with other services, and follow-ups when working with individuals with pnOCD Heath professionals should consider the effect on the infant if the parent is very anxious, and how this may affect the parent–child relationship and interactions Health professionals should make adaptations due to the perinatal period (e.g., schedule appointments around the infant routine, modify homework, discuss ahead of time challenges that may occur as the infant matures) where possible Health professionals should be mindful of the health and welfare of both the parent/s and the fetus/infant.^a^ Health professionals should consider the individual's available resources, such as energy, time, money, motivation and support networks, as well as available resources of significant others Health professionals should encourage bonding between the parent with pnOCD and the infant (e.g., encourage attentive feeding, play) where possible to reduce pnOCD related avoidance Health professionals should be aware of the availability of hospital mother-baby units if an infant is under 12 months of age or adult mental health inpatient admission in severe circumstances of pnOCD Psychoeducation resources should be offered to the individual at all stages of care When PnOCD psychoeducation resources are provided by health professionals, the parent should have the opportunity to ask questions regarding the information in a safe environment Treatment planning should involve consultation with other health professionals providing ongoing care (e.g., General Practitioner, Psychologist or Psychiatrist, Midwives, Obstetrician, Nurses) When advice is offered on treatment options, health professionals should inform consumers that current evidence-based treatments for OCD are also effective for pnOCD Once a diagnosis of pnOCD has been made, symptoms should be monitored on a regular basis, with frequency of monitoring dependent on severity If the assessing clinician is unable to provide ongoing treatment, the individual should be referred for ongoing treatment with an appropriate mental health professional.^a^ The individual with pnOCD should be informed of all referrals and the reason behind them PnOCD treatment referrals should be followed up within a week The individual with pnOCD should be consulted when considering changing the type or increasing or decreasing the frequency and intensity of professional supports Care should be continued until the individual and treating professional both agree it is time to cease.^a^ If the individual declines pnOCD treatment and there are no risk factors, parents should be provided with information regarding how to seek services in the future should they choose to.^a^
Treatment	If the parent is experiencing sub-clinical pnOCD symptoms that are not currently causing significant distress or impairment, clinicians should provide psychoeducation about pnOCD (including when treatment would be indicated); and provide the parent information about accessing appropriate services should they chose to do so in future Treatment options for pnOCD should be offered at all levels of the healthcare system with intensity of care dependent on the level of symptom severity, distress, and impairment that the parent is experiencing Clients should be made aware of support groups and resources to assist them in further understanding the disorder Clinicians should be aware that psychological and pharmacotherapy treatment options exist for treating pnOCD, both with empirical evidence supporting their efficacy.^a^ Clinicians should be aware that combined therapy including both pharmacotherapy and CBT may be more effective than monotherapy (i.e., pharmacotherapy or CBT alone) for some individuals with pnOCD Individuals experiencing pnOCD should be offered cognitive behaviour therapy (CBT) that includes exposure and response prevention (ERP) as a core component as a first-line treatment option Clinicians should be aware that CBT for pnOCD can be effectively delivered on either an individual basis or in a group Treating clinicians should safely involve the infant in CBT (e.g., during exposure-based exercises) if the theme of obsessions/compulsions relates to the infant When parents request forms of psychotherapy (e.g., psychoanalysis or hypnosis) other than cognitive and behavioural therapies, they should be informed there is currently insufficient evidence to support such methods in treating pnOCD Treatment planning and implementation should target relevant, individual factors (e.g., cognitions and behaviours, interpersonal distress) that are maintaining the parent’s pnOCD symptoms and the associated impacts Clinicians should consider the impact of any psychosocial issues that may impact the capacity for parents to engage in evidence-based treatments for pnOCD (e.g., social support, interpersonal concerns, financial means) when treatment planning Where parents are experiencing comorbidities alongside pnOCD, clinicians should recommend the treatment with the greatest potential for reduction of distress and improvement in functioning Where a parent with pnOCD is also experiencing significant comorbid psychiatric disorders or psychosocial issues, a multi-disciplinary approach to care should be considered The treatment plan for pnOCD should be jointly agreed by the parent and clinician, after the parent has been provided with information about what various treatment options may involve and the evidence supporting their use Where possible, advice should be sought from an experienced perinatal psychiatrist regarding pharmacological treatment for pnOCD Clinicians prescribing psychotropic medication should consider the mother's preferences regarding pregnancy/infant feeding in making decisions on pharmacological treatment for pnOCD Clinicians should inform parents with pnOCD about relative risks of side-effects of pharmacotherapy for a woman and the risks in pregnancy and/or breastfeeding. This needs to be balanced against the risks of not taking the recommended treatment Written information about the benefit and side effect profile of recommenced or prescribed medications should be provided wherever possible Selective Serotonin Reuptake Inhibitors (SSRIs) or Tricyclic Antidepressants (TCAs) should be considered as a recommended pharmacological treatment option for pnOCD, dependent on the parent’s treatment goals and preferences, symptom severity, psychiatric and medical history, and the presence of any comorbid medical or psychiatric disorders (e.g., mood disorders), and taking risk of overdose, and implications for pregnancy/breastfeeding into account Pharmacological treatment should be reviewed regularly by a prescribing clinician When parents request non-pharmacological biomedical therapies (electroconvulsive therapy, transcranial magnetic stimulation, deep brain stimulation, psychosurgery) they should be informed that there is currently mixed evidence to support such methods in treating pnOCD Clinicians should consider the need for inpatient care where pnOCD symptoms require intensive intervention that is best provided within inpatient care, where there are significant concerns about the impact of pnOCD on parenting and the parent-infant relationship, or where there are concerns about the parent’s immediate risk of suicide or significant self-harm When pnOCD inpatient care is provided, where possible a mother baby unit admission should be sought so the baby can remain in the mother’s care Crisis resources (e.g., hospital admission, crisis helplines) should be offered to people who disclose they are experiencing pnOCD symptoms If there is an inadequate response to either pharmacotherapy alone (by 12 weeks) or CBT alone (more than 10 therapist hours), the treatment plan should be reviewed Provisions should be made during treatment programs to include the child’s presence and allow the parent to attend to the child’s needs (e.g., feeding, changing) Following completion of treatment, pnOCD symptoms should be reviewed regularly by a clinician for a period of at least 12 months Following completion of treatment, pnOCD symptoms should be reviewed regularly by a health professional for a period agreed on by the clinician and parent Clinicians offering psychological treatment for pnOCD should have received formal specialist training and clinical supervision in the treatment of OCD Clinicians who have not undertaken specialist training and clinical supervision in the treatment of OCD should refer the parent to an appropriately qualified treatment provider Clinicians offering psychological treatment for pnOCD should engage in ongoing professional development and clinical supervision in the treatment of OCD
Partners and Families	The individual with pnOCD should be asked about whom from their family they would like to be involved in their care Parents and significant others should be provided with psychoeducation about pnOCD (including about intrusive thoughts), the nature and goals of treatment, and advice on how to best support the person experiencing pnOCD to engage in treatment Parents and significant others should be offered the opportunity to be involved in treatment if the individual with pnOCD consents, and it is considered that this involvement would benefit the consumer Family accommodation, where family members inadvertently contribute to the maintenance of OCD through efforts to help (e.g., providing excessive reassurance, assisting with ritual completion or avoidance of situational triggers), should be explained to family members and incorporated into pnOCD treatment planning
Culture and Diversity	Assessment and treatment of pnOCD should be offered to all parents where relevant, regardless of if they are the biological parent, their sex, gender identity, sexual identity, age, race, cultural beliefs, and religious beliefs Treatment and assessment of pnOCD should take into account the individual's cultural and/or religious beliefs where the consumer believes this to be relevant to their care Treatment should be adjusted to accommodate cultural or religious concerns, where possible. If accommodations cannot be made, the risks of electing not to proceed with the recommended treatment approach should be discussed

^a^Statements endorsed by 100% of consumer and professional panellists as ‘Important’ or ‘Essential’ to include as recommendations

### Data analysis

**Fig. 1:**
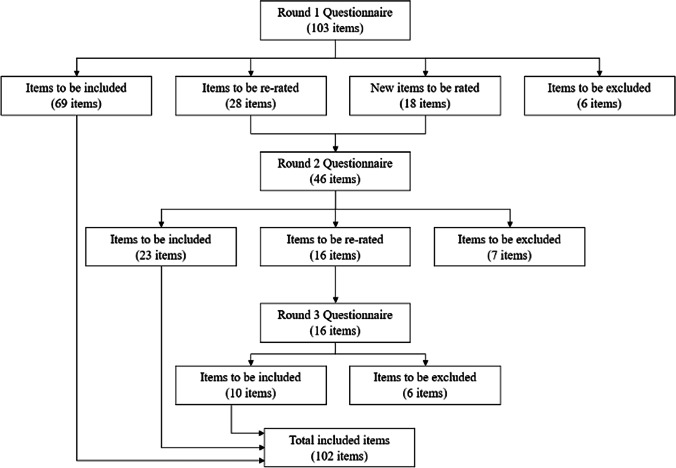
Number of items included, re-rated and excluded at each round of the study

One-hundred and three statements across eight topic areas were rated in Round 1. Comments from panellists in Round 1 contributed a further 18 statements that were included in Round 2. Figure 1 indicates the number of items which were included, retained, and excluded for re-rating during each round.

## Results

### Endorsed items

From a total of 121 statements, 102 were endorsed; 69 from Round 1, 23 from Round 2, and 10 from Round 3. Six of the 18 participant-generated recommendations received endorsement. A list of the endorsed statements is provided in Table 2. Of note, eleven statements (10.8% of endorsed statements) were unanimously endorsed by all members of both panels as ‘essential’ or ‘important’ to include (see Table 2).

### Excluded items

Nineteen statements were excluded; six from Round 1, seven from Round 2, and six from Round 3. These excluded statements are available in the supplementary materials. Certain statements were definitively rejected by both the Professional and Consumer Panels. Items with the lowest endorsement rate across both panels included the statement that “*The assessing clinician should also provide the pnOCD treatment”* and *“Benzodiazepines and psychoeducation should be considered in some pnOCD cases as an initial treatment approach, instead of SSRI’s or other medications”.* Some statements that were suggested by panellists in Round 1were also rejected strongly by both panels in the following round (e.g., “*Other psychotherapies should be offered (e.g., Jungian-Feminist Therapy) as options for treatment”*). Finally, some statements were excluded due to disagreement between panels.

*Differences between consumer and professional panels.* Post hoc analysis was conducted to explore any differences between Professional and Consumer Panels’ endorsement of recommendations throughout the three stages of the study. Overall, there was substantial agreement Consumers were less likely to endorse items in the first round compared to the Professional Panel, although this difference appeared to diminish in subsequent rounds (see Table 3). The results within the Consumer Panel were also more varied in the first round than within the Professional Panel whereby consumers had a greater number of items to be re-rated. Again, these differences appeared to decrease in subsequent rounds. between the panels, with over 75% agreement in Round 1, and over 65% agreement in Round 2. Statements were deemed to have considerable disagreement if the difference in endorsement across panels was ≥ 30% (Rosenthal 1996). Only five items met the criteria for considerable disagreement, as shown in Table 4. Consumers were more likely to rate those statements with large differences as important or essential than the professional panel.

**Table 3:** Summary of items endorsed, re-rated, or rejected by consumers, professionals and by both panels combined for rounds one, two, and three

Survey Round	Panel	Endorsed (>80%)	Re-rated (70% - 79% or endorsed by only one panel)	Rejected (< 70%)
Round 1	Consumers	76	17	10
	Professionals	89	8	6
	*Both Panels*	*69*	*28*	*6*
Round 2	Consumers	29	9	8
	Professionals	29	9	8
	*Both Panels*	*23*	*16*	*7*
Round 3	Consumers	13	N/A^a^	3
	Professionals	11	N/A^a^	5
	*Both Panels*	*10*	N/A^a^	*6*

^a^N/A: Not Applicable. Round 3 did not allow for items to be re-rated

**Table 4:** Statements with large differences (≥ 30%) in endorsement between panels

Statement	Endorsement level by Professionals Expert Panel (%)	Endorsement level by Consumers Panel (%)	Difference (%)
Clinicians should determine whether any taboo thoughts present are experienced as ego-syntonic (consistent with the parent’s beliefs, desires and wishes) or ego-dystonic (inconsistent with the parent’s beliefs, desires and wishes and therefore experienced as senseless, unwanted and intrusive)	100	64.3	35.7
Individuals experiencing pnOCD should be offered mother-infant therapy, or peer support/interpersonal therapy groups, as an adjunct therapy to evidenced-based treatment	40	78.6	38.6
Parents should have the option to speak to someone who has had lived experience of perinatal OCD.^a^	8.3	90	81.7
Assessments for pnOCD should include questions regarding significant others’ beliefs about mental health in the perinatal period.^a^	25	80	55
Documentation of specific PnOCD symptoms should be kept confidential and only shared with those clinicians who are pnOCD specialists to prevent stigma and overprotective treatment.^a^	8.3	50	41.7

Endorsement is based on the percentage of ‘essential’ or ‘important’ ratings

^a^Statement added from participant suggestions

## Discussion

The aim of the current study was to address the gap in literature regarding the clinical management of pnOCD; more specifically, to develop recommendations for the assessment, treatment, and management of pnOCD, and other considerations to best support individuals with this disorder. To our knowledge, this is the first study to systematically collate expert views, and generate best-practice recommendations for pnOCD, using a consensus-based methodology.

 Results indicated that participants agreed with most (96/103) of the initial recommendation statements which were created based on the initial literature review and panelist suggestions. Of particular significance is the high degree of agreement between the Professional and Consumer Panels as to which statements should be endorsed, which may confer confidence as to the veracity of the final recommendation set. A strength of the Delphi method is that participants can provide input about amendments to statements, or additional statements which they think should be included (Keeney et al. 2010). This process resulted in the endorsement of an additional six practice recommendations generated by professionals or consumers, therefore adding the comprehensiveness of the final recommendation set and highlighting the importance of including consumers in the creation of such recommendations.

Most items that were endorsed by less than 70% of consumers fell within the ‘Differential Diagnosis’ and ‘Case Care Considerations’ categories. Post hoc analysis identified that these items were not rated as ‘unimportant’ by the consumers, but as ‘don’t know/depends’. This likely shows the Consumer Panel’s ability to acknowledge the limits of understanding in these areas and, therefore, a preference not to make judgment. A theme of statements which were rejected by either Panel were those relating to child safeguarding. The Professional Panel’s rejection of such statements may be due to child safeguarding being a challenging decision for health practitioners to make, requiring careful consideration of risks of both direct and indirect infant harm, often in the context of comorbid maternal mental health diagnoses (i.e., Poinso et al. 2002). It is likely that Consumers rejected recommendations regarding child safeguarding given the potential risk of pnOCD symptoms being misidentified as indicating actual risk of harm to the infant, which may prevent parents from disclosing pnOCD symptoms to health practitioners from the outset (Megnin-Viggars et al. 2015). Regardless, these differences in professional and consumer perspectives highlight the potential consequences of the mischaracterisation of pnOCD, and the need for targeted guidance to increase clinicians’ understanding of how to assess and manage pnOCD.

This study has made a unique and much needed contribution to the pnOCD literature. Existing OCD and perinatal mental health best-practice guidelines do not detail specific considerations relevant to perinatal OCD which are important to explore (American Psychiatric Association, 2007a,b; Austin and Highet 2017; beyondblue 2011; National Institute for Health and Care Excellence, 2005, 2014). For example, the importance of assessing whether any intrusive thoughts of harming the baby are experienced as ego-syntonic or ego-dystonic, which is central to distinguishing between infant harm-related thoughts in OCD or psychosis (Fairbrother & Abramowitz 2016). This is particularly important given that parents’ may be reluctant to disclose intrusive thoughts for fear they will be misunderstood by healthcare providers (Challacombe & Wroe 2013), specific treatment indications for OCD (e.g., exposure and response prevention; Stein et al. 2010), and the potentially aggravating effects of misdiagnosis or mistreatment of pnOCD (Challacombe & Wroe 2013; Gershkovich 2003; Mulcahy et al. 2020; Storch 2015; Veale et al. 2009).

A strength of this study is that the endorsed statements provide guidance that spans the need for prevention via psychoeducation through to complex differential diagnostic and treatment considerations. By having participants rate, rather than rank, whether an item is important for inclusion in the guidelines, we have identified a comprehensive list that does not prioritise any one aspect of assessment, treatment or management over another. Each item may therefore be considered important both in isolation and in conjunction with the other items listed. For example, the provision of psychoeducation “…that there is no evidence that parents experiencing harm-related intrusions, no matter how horrific in content, will act on these thoughts” is important corrective information for someone experiencing pnOCD intrusions. However, it is equally as important to appropriately assess the client and follow the clear differential diagnosis statements to ensure the person is presenting with pnOCD and not another mental health disorder warranting a different treatment and management approach.

Certain limitations of the Delphi methodology used in this study must be acknowledged. First, five members of the Professional Panel and four members of the Consumer panel did not complete Round 1 of the study. A possible reason for this is that Round 1 included 103 items which may have been too time-demanding for some panellists to complete (Keeney et al. 2010; van Zolingen & Klaassen 2003). This may have particularly been the case given that this research was conducted during the unprecedented time of the initial COVID-19 global pandemic outbreak. However, there were still enough participants in each round as part of a homogeneous panel for results to be deemed representative of the larger population and therefore generalisable (Keeney et al. 2010). The relative similarity in the size of both the final Professional and Expert Panels also means that the views of both groups were equally weighted in reaching the study findings. Second, this study was focused on mothers with OCD, although fathers are also known to experience OCD in the perinatal period (Abramowitz et al. 2001). Thus, the guidelines developed in the present study may not apply to fathers with pnOCD, and there is a critical need for further research in this area more broadly. Finally, the specificity of recommendations was somewhat impacted by the global recruitment of experts, and the recruitment of both consumer and professional expert panels. For example, the lack of availability of mother-baby units in some locations led to the recommendation that “Health professionals be aware of the availability” of such services, rather than specifying that individuals should be referred to a mother-baby unit. Furthermore, we did not consider that consumers would likely had sufficient training to make a valid judgement on the psychometric properties of screening or assessment tools to recommend any particular one over another.

Given the paucity of pnOCD literature, this set of consensus findings may be a useful basis for identifying gaps in the pnOCD literature. For example, we now know that both consumers and experts agree with the need for routine screening via pnOCD specific screening items, but there is a need to empirically evaluate the existing screening tools to determine a preferred tool. In sum, the current study represents a novel contribution to the emerging clinical literature on pnOCD and offers an important step forward in efforts to develop and disseminate best-practice for pnOCD.

## Supplementary information

Below is the link to the electronic supplementary material.

Supplementary file1 (DOCX 16 KB)

## Data Availability

The data comprising the results of this study are available upon reasonable request from the corresponding author, MM.
