# Supplementary material for: Consensus recommendations for the assessment and treatment of perinatal obsessive–compulsive disorder (OCD): A Delphi study
Source: Arch Womens Ment Health. 2023 May 3;26(3):389–99. doi: 10.1007/s00737-023-01315-2 (PMC10155656; doi:10.1007/s00737-023-01315-2)
Supplement: Supplementary file 1 — Supplementary file1 (DOCX 16 KB) [file 737_2023_1315_MOESM1_ESM.docx]

**Supplementary materials**

*List of Excluded Statements, by Topic.*

| Topic | Statement |
| --- | --- |
| Psychoeducation | Psychoeducation should be given about the commonalities and differences between PnOCD symptoms and perfectionism around parenthood. |
|  | Psychoeducation should only be provided when there is a clear diagnosis of perinatal OCD. |
|  | Parents should be informed that intrusive thoughts are common but often settle within the first 6 weeks postpartum. |
|  | Clinicians should describe PnOCD symptoms as commonly occurring but should not refer to the “normalising” of symptoms. |
|  | Parents should have the option to speak to someone who has had lived experience of perinatal OCD. |
|  | Caution should be exercised when discussing PnOCD symptoms and impacts (e.g. on the mother-infant relationship) so as not to further increase the parent’s anxiety. |
| Screening | When screening for PnOCD, no more than one question should be used. |
|  | Assessments for pnOCD should include questions regarding significant others’ beliefs about mental health in the perinatal period. |
| Assessment | Documentation of specific PnOCD symptoms should be kept confidential and only shared with those clinicians who are PnOCD specialists to prevent stigma and overprotective treatment. |
|  | Parents should not be asked directly about intrusive thoughts due to the potential for this to be perceived as being quite aggressive and potentially threatening - the person may not feel able to be honest. |
| Differential Diagnosis | Clinicians should determine the parent’s psychological response to any taboo thoughts present, with distress during and after the thought being indicative of pnOCD, but excitement, arousal, agitation or relief during the thought as being indicative of other mental health concerns. |
|  | Clinicians should determine the parent’s physiological response to any taboo thoughts present, with high fear responses both during and after the thought as being indicative of pnOCD. |
|  | If it is uncertain whether the diagnosis is pnOCD, referral for child safeguarding should consider the balance of risks of referral versus non-referral to the child and parent, local professional reporting requirements, and professional practice standards. |
| Case Care Consideration | Referral for child safeguarding should be considered when the pnOCD symptoms are impacting on the parent’s capacity to maintain their parental responsibilities. |
|  | The assessing clinician should also provide the pnOCD treatment. |
| Topic | Statement |
| Treatment | Benzodiazepines and psychoeducation should be considered in some PnOCD cases as an initial treatment approach, instead of SSRI’s or other medications. |
|  | Other psychotherapies should be offered (e.g., Jungian-Feminist therapy) as options for treatment |
|  | Individuals experiencing pnOCD should be offered mother-infant therapy, or peer support/interpersonal therapy groups, as an adjunct therapy to evidenced-based treatment. |
| Partners and Families | Healthcare professionals need to ensure that other dependent children are able to be cared for by the spouse or other support networks while the individual with pnOCD is attending inpatient/outpatient treatment to facilitate engagement and reduce attrition. |
